# The genome of antibiotic-producing colonies of the Pelagophyte alga *Chrysophaeum taylorii* reveals a diverse and non-canonical capacity for secondary metabolism

**DOI:** 10.1038/s41598-023-38042-8

**Published:** 2023-07-24

**Authors:** Jack R. Davison, Rahim Rajwani, Gengxiang Zhao, Carole A. Bewley

**Affiliations:** 1 Laboratory of Bioorganic Chemistry, National Institute of Diabetes and Digestive and Kidney Diseases, National Institutes of Health, Mailstop 0820, Bethesda, MD 20892 USA; 2 Present Address: LifeMine Therapeutics, 30 Acorn Park Dr., Cambridge, MA 02140 USA

**Keywords:** Biosynthesis, Natural products, Genomics, Antibiotics

## Abstract

*Chrysophaeum taylorii* is a member of an understudied clade of marine algae that can be responsible for harmful coastal blooms and is known to accumulate bioactive natural products including antibiotics of the chrysophaentin class. Whole genome sequencing of laboratory-cultivated samples revealed an extensive and diverse complement of secondary metabolite biosynthetic genes in *C. taylorii*, alongside a small microbiome with a more limited biosynthetic potential. 16S microbiome analysis of laboratory cultured alongside wild-collected samples revealed several common taxa; however, analysis of biosynthetic genes suggested an algal origin for the chrysophaentins, possibly via one of several non-canonical polyketide synthase genes encoded within the genome.

## Introduction

Pelagophyte algae are a taxonomic group of heterokontophyte or microalgae distributed globally and across diverse marine ecosystems. Members of this group have been observed in coastal waters from the Caribbean^1^ and Western Pacific^2^ to the Mediterranean^3^ and are receiving increased attention due to the ecological effects they can exert on marine environments. *Chrysophaeum taylorii* is a colony-forming species of the Pelagophyceae class of stramenopiles algae^4^. Morphologically, Pelagophyte algae vary widely, from motile unicellular organisms to colonial multicellular assemblies with cells embedded in secreted mucilage, such as *C. taylorii*^5^. *C. taylorii* is considered by some to be a nuisance organism due to its ability to form large mucilaginous blooms in favorable conditions, outcompeting other benthic organisms. In recent decades, *C. taylorii* has been observed with increasing frequency in Mediterranean coastal waters, finding conditions including water depth, substratum characteristics, and hydrodynamics that are conducive to extensive growth^6,7^. Importantly, several other pelagophytes are prominent bloom-forming species; for example Pelagomonas and Pelagococcus are responsible for brown tides in open oceans, while *Aureococcus anophagefferens* and *Aureoumbra lagunensis* form harmful algal blooms in coastal environments^8^. Bloom-forming pelagophytes have been shown to exhibit strong allelopathic effects on the growth of other bacterial and eukaryotic competitor species, facilitating rapid expansion in their environment^9^.

Several biologically active secondary metabolites (or natural products) have been isolated from *C. taylorii* samples, including hormothamnione^10^ and the chrysophaentin family of antibiotics^11^. The chrysophaentins are promising candidates as new antibiotics with a novel mechanism of action targeting the bacterial cytoskeletal protein FtsZ^12^. However little is known about the biosynthetic pathways that are required for the production of secondary metabolites such as the chrysophaentins in *C. taylorii* or related algae. The chrysophaentins represent a new scaffold within the the bisbibenzyl class of natural products that are typically associated with Bryophyte non-vascular land plants; for example, the marchantins that are produced by members of the genus *Marchantia*^13^.

Despite the importance of the pelagophyte group of algae genomic data are only available for three species in the clade to date. These include the single-celled phytoplanktonic *A. anophagefferens*^14^, *Pelagococcus subviridis*^15^, and *Pelagomonas calceolata*^16^. There are no genome-level data available for pelagophyte species that form higher-order colonial assemblies, including *C. taylorii*. A lack of published genomic information is a limitation for comparative studies of important features of this group of algae, including their morphological, bloom-forming, and biosynthetic capabilities. Here, we sequenced the genome and microbiome of *C. taylorii* samples that produce chrysophaentins, grown under laboratory conditions, with the goal of identifying conserved pathways that may control antibiotic biosynthesis in this system.

## Results

### Genome assembly and multi-locus phylogenetic placement of *C. taylorii*

Laboratory cultures of *C. taylorii* NIES-1699 were processed for DNA sequencing using short read (Illumina HiSeq) and long read (Oxford Nanopore and Pacific Biosciences) technologies (Supplementary Table S1). Assembly of the combined dataset (108 Gbp) led to the discovery of the algal mitochondrion (58 kbp), chloroplast (104 kbp) and 12 single-contig bacterial genomes (Supplementary Table S2, S3). After refinement, the final *C. taylorii* chromosomal contig set contained a total of 71 Mbp sequence on 702 contigs, with an N50 of 157 kbp (Table 1). BUSCO (Benchmarking Universal Single-Copy Orthologs) analysis of the *C. taylorii* assembly identified 81.5% conserved single copy genes from the Eukaryota set, indicating favorable completeness of the genome, comparable to the NCBI reference genome of the related pelagophyte alga *Aureococcus anophagefferens* (GCA_000186865.1: 76% complete Eukaryota BUSCOs).

**Table 1:** Taxonomic assignment and assembly statistics of contigs constructed from Illumina short read and long read datasets.

Taxonomic assignment	Size/Mbp	N50 (Illumina)/kbp	N50 (Long read)/kbp	% BUSCOs
*Alcanivorax jadensis*	3.6	392	n/a	100.0
*Altererythrobacter ishigakiensis*	2.6	2636	2636	98.4
*Ekhidna lutea*	4.3	738	4266	97.6
*Tepidicaulis sp.*	3.9	3361	3900	99.2
*Phyllobacteriaceae sp.*	3.4	2008	3439	99.2
*Planctomycetes sp.*	3.2	2375	3234	84.7
*Roseibium album*	6.4	n/a	6416	100.0
*Roseibium sp.*	5.3	n/a	5348	99.2
*Reichenbachiella sp.*	5.1	n/a	5075	97.6
*Balneola sp.*	3.6	n/a	3641	96.0
*Hyphomonas. beringensis*	3.6	n/a	3584	92.7
*Marinobacter nauticus*	4.4	n/a	4024	93.5
*Crocinitomicaceae sp.*	3.7	n/a	3469	83.1
*C. taylorii*	71.0	12.4	157	81.2
*C. taylorii* chloroplast	0.10	104	104	n/a
*C. taylorii* mitochrondrion	0.06	58	58	n/a

**Figure 1:**
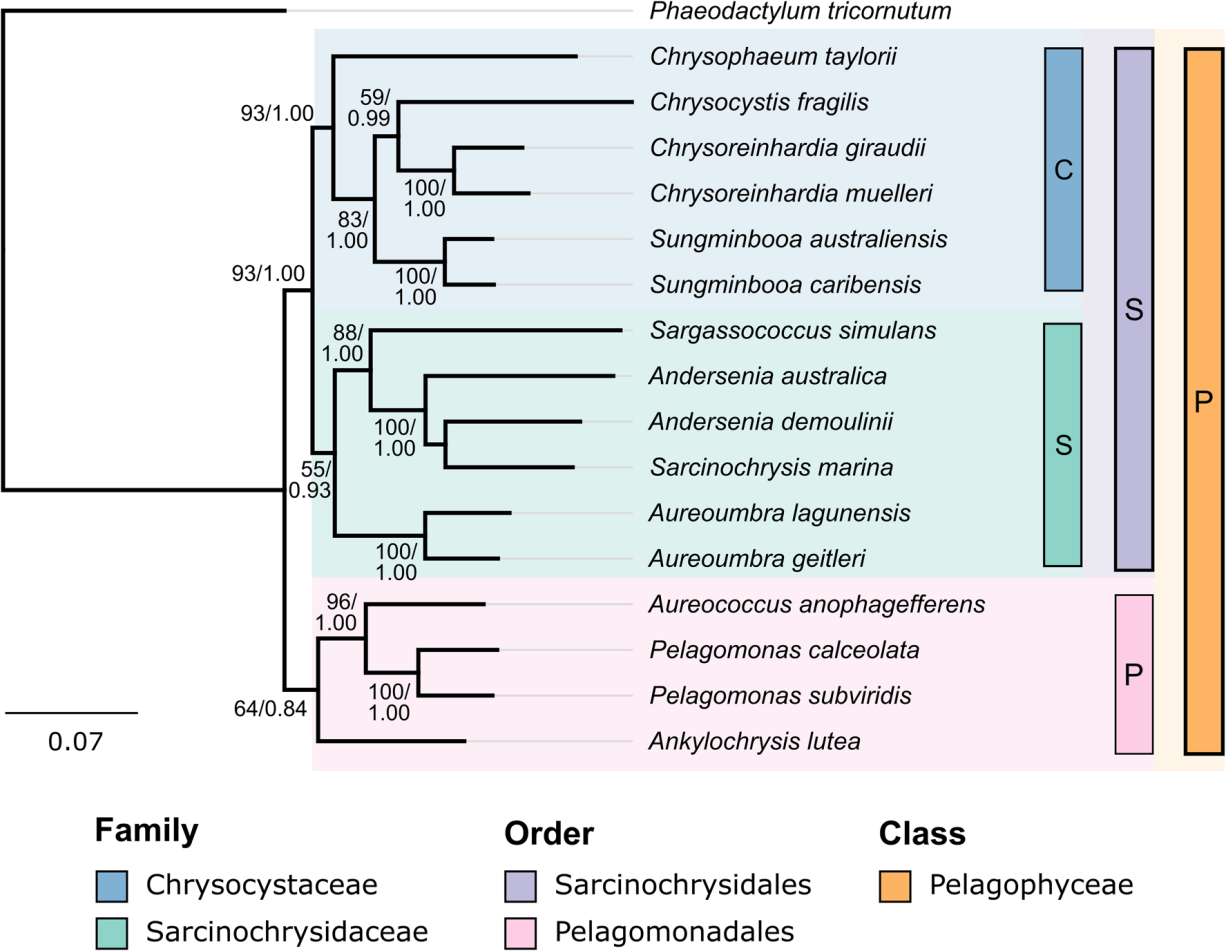
*Phylogenetic placement of C. taylorii.* Inferred maximum likelihood phylogenetic tree of a multilocus alignment of *C. taylorii* sequence, alongside other known members of the Pelagophyceae class of algae, rooted on the outgroup diatom *Phaeodactylum tricornatum*. The class is divided into two orders—Sarcinochrysidales and Pelagomonadales, while the Sarcinochrysidales order consists of two families—Chrysocystaceae and Sarcinochrysidaceae. Class, orders and families shown as vertical bars (right). *C. taylorii* is assigned to the Chrysocystaceae family. Scale bar represents substitutions per site, clade support values labelled—bootstrap %/posterior probability.

*C. taylorii* has previously been assigned taxonomically to the Pelagophyceae class of heterokont algae, within the order Sarcinochrysidales^4^. Sarcinochrysidales consists of algae that form multicellular colonial structures, including filaments, sheets, and amorphous assemblies^17^. The most recent multilocus phylogenetic analyses of the Sarcinochrysidales split the order into two families, Chrysocystaceae and Sarcinochrysidaceae^18^. Analysis of *C. taylorii* using 18S rRNA, psaA, psaB, psbA, psbC and rbcL gene sequences placed it within the Chrysocystaceae family, alongside the genera *Chrysocystis*, *Chrysoreinhardia*, and *Sungminbooa* (Fig. 1).

### Gene annotation and analysis

The *C. taylorii* long-read contig set was annotated for coding sequences using transcript evidence from RNA-seq reads alongside protein homology evidence, resulting in a total of 10,810 predicted genes (Supplementary Figure S1). This gene set contained 81.5% of eukaryotic BUSCOs, indicating that it was fully representative of the BUSCO complement identified from the *C. taylorii* nucleotide assembly (81.2%).

To analyze the secondary metabolic potential of *C. taylorii*, annotated gene predictions were used as input for antiSMASH 6.0.0. The antiSMASH pipeline uses Hidden Markov Model profiles to identify and annotate genes functionally associated with secondary metabolism^19^. In the *C. taylorii* genome antiSMASH identified a total of 26 core secondary metabolic genes, predominantly polyketide synthases (PKS) or non-ribosomal peptide synthases (NRPS). Within candidate cluster regions, antiSMASH identified at most one core gene per region with an average of 1 tailoring gene, indicating a lack of biosynthetic gene clustering (Supplementary Figure S2) (Supplementary Table S4). In contrast, there were up to five biosynthetic core genes in the BGCs of the *C. taylorii* microbiome surrounded by an average of 4 tailoring genes (Supplementary Table S4). This was also found to be consistent with reanalysis of cyanobacterial PKS and NRPS regions from the BiGFAM database where the average number of genes ranged from 31 to 65 across region types (Supplementary Figure S3). This low level of colocalized biosynthetic genes suggests that clustering of secondary metabolic genes is not a major feature in *C. taylorii,* making it challenging to identify related genes in biosynthetic pathways by association.

**Figure 2:**
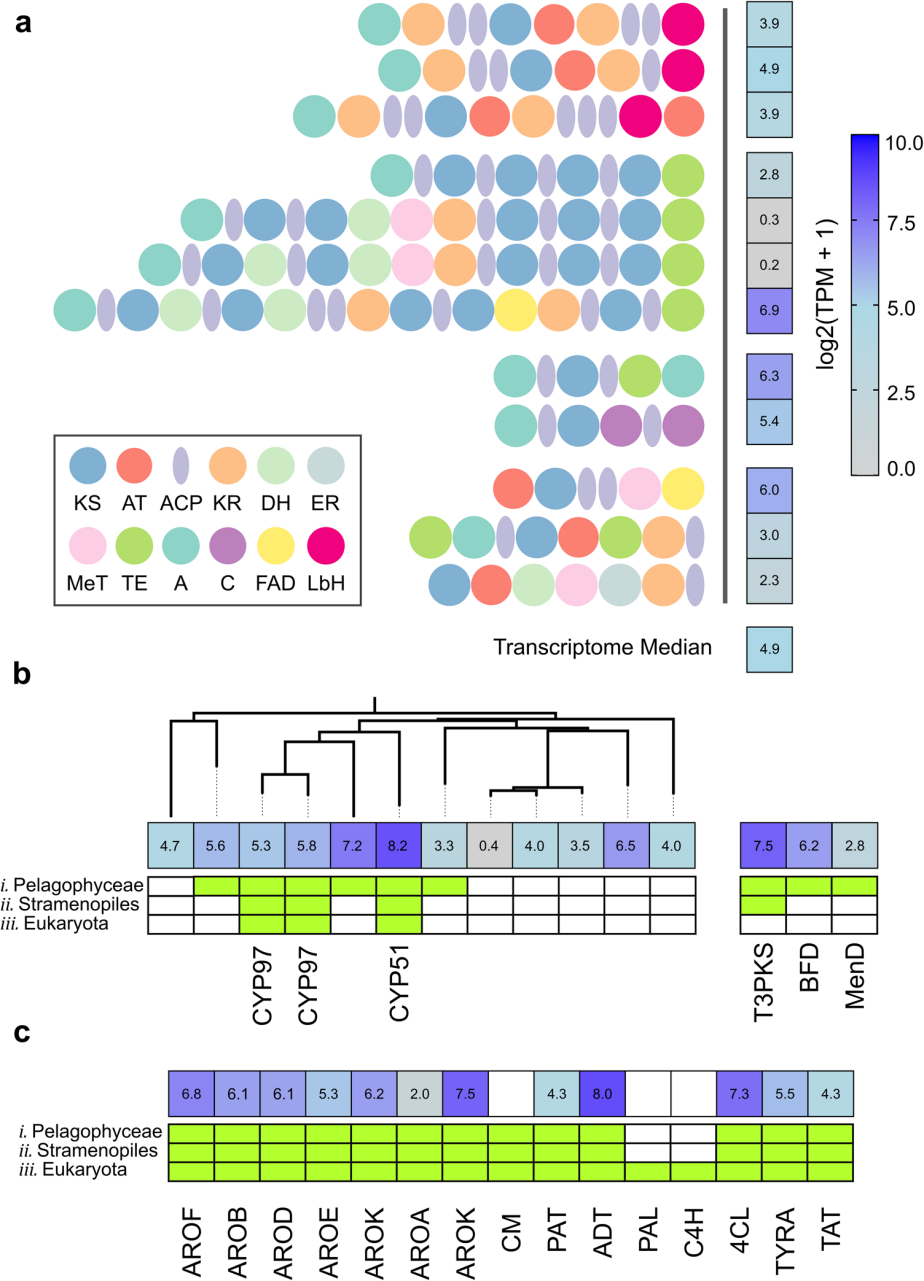
Secondary metabolite genes in *C. taylorii*. (**a**) Domain structures of 12 type I PKS genes identified in the *C. taylorii* genome assembly, annotated with RNA-seq-derived normalized expression level per gene (log2 transcripts per million). Expression level of the median of the transcriptome is provided for reference. Domains utilized in *C. taylorii* PKS genes are ketosynthase (KS), acyltransferase (AT), acyl carrier protein (ACP), ketoreductase (KR), dehydratase (DH), enoylreductase (ER), methyltransferase (MeT), thioesterase (TE), adenylation (A), condensation (C), FAD-dependent oxidoreductase (FAD), and left-handed beta helix (LbH). (**b**) Expression level of 12 cytochrome P450 sequences identified in the *C. taylorii* genome sequence, alongside a type III PKS (T3PKS) and two acyloin synthases (homologs of BFD and MenD). Green bars indicate whether a putative ortholog was identified in the genome of *i*) the pelagophyte alga *A. anophagefferens*, *ii*) either of the stramenopiles algae *P. tricornatum* or *Ectocarpus siliculosis,* or *iii*) the plant *Arabidopsis thaliana*. (**c**) Expression levels and orthology of genes identified from the shikimate and cinnamate pathways in *C. taylorii*.

*C. taylorii* has a large potential for polyketide biosynthesis, with 13 distinct PKS genes identified (Fig. 2a). *C. taylorii* PKS genes were highly divergent from existing fungal and bacterial systems, incorporating atypical domains and featuring non-canonical domain arrangement. The largest category of PKS genes in *C. taylorii* consists of modular PKS missing an integral acyl-transferase domain (trans-AT)^20^, with modules varying from a simple ketosynthase (KS) domain to a suite of reductive and methylation functionalities. All genes of this type encoded an atypical N-terminal adenylation-like domain expected to load an advanced starter unit^21^. The remaining PKS genes contained AT domains, and appeared to be iterative, each containing a single KS domain. Several atypical domains such as FAD-dependent oxidoreductase or left-handed beta helix LbH^22^ were identified as part of these synthases. RNA-seq analysis revealed that five of the PKS genes were expressed at equal to or greater than the median expression level of the transcriptome (Fig. 2a). Given that chrysophaentins were abundant in all samples analyzed, it was expected for the genes involved in their biosynthesis to be strongly and consistently active. Genes with high transcript counts, such as the five PKS genes indicated, are potential candidates for chrysophaentin biosynthesis. Besides the type I PKS genes, one type III PKS was identified in the genome.

Non-ribosomal peptide synthase (NRPS) genes were also common, with nine identified. Additionally, three terpene synthase genes were found, and two thiamine pyrophosphate (TPP) dependent acyloin synthases (with homology to benzoylformate decarboxylase BFD, and the menaquinone biosynthetic enzyme MenD, respectively). The PKS and NRPS complement of *C. taylorii* was greatly expanded compared to its closest sequenced relative *A. anophagefferens* for which a total of five core genes were identified by antiSMASH analysis, which may reflect an expanded capability for production of secondary metabolites in *C. taylorii*, including the chrysophaentins.

Besides PKS and NRPS genes, a common gene class associated with secondary metabolism is the cytochrome P450. Twelve genes of this family were identified in the *C. taylorii* genome (Fig. 2b), of which three could be assigned to the known P450 clans CYP97 and CYP51 which are common to stramenopiles algal genomes^23^. Six of the P450 genes had putative orthologs in the related *A. anophagefferens* genome, and the remaining six were unique to *C. taylorii*, suggesting they may be involved in specialized metabolism within this species. RNA-seq analysis showed that five of the six specialized P450 genes were expressed in chrysophaentin-producing conditions, making them possible candidates in chrysophaentin biosynthesis.

Aromatic natural products such as chrysophaentin can be derived from intermediates in the shikimate pathway^24^. *C. taylorii* contains most of the genes from this pathway (Fig. 2c) but appears to be lacking genes encoding several elements including chorismate mutase (CM), phenylalanine ammonia-lyase (PAL), and cinnamate-4-hydroxylase (C4H). PAL and C4H are commonly missing in stramenopiles algae^25^, though several are known to produce cinnamic acid and derivatives^26,27^. This observation raises the possibility that alternative enzymes may exist to produce cinnamate, as proposed in bacteria such as *Nannocystis pusilla*^28^, or that synthesis could be complemented by mutualistic interactions^29^.

One of the distinguishing features of chrysophaentins is polyhalogenation. It was hypothesized that a vanadium chloroperoxidase might catalyze this reaction as reported for other halogenated natural products from marine algae^30^. However, no homolog of vanadium chloroperoxidase was observed in the genome (Supplementary Table S5). We extended our analysis by including all six major families of halogenases mapping to 13 Pfam or InterPro domains (Supplementary Table S5)^31^, but did not identify any new significant candidates. Several genes encoding domains for large superfamilies with some halogenase members were identified, including alpha/beta hydrolase fold, PAP2 superfamily and Phytanoyl-CoA dioxygenase. This analysis suggests that an atypical halogenase might be responsible for halogenation in *C. taylorii*.

### Microbiome of *C. taylorii* colonies

**Figure 3:**
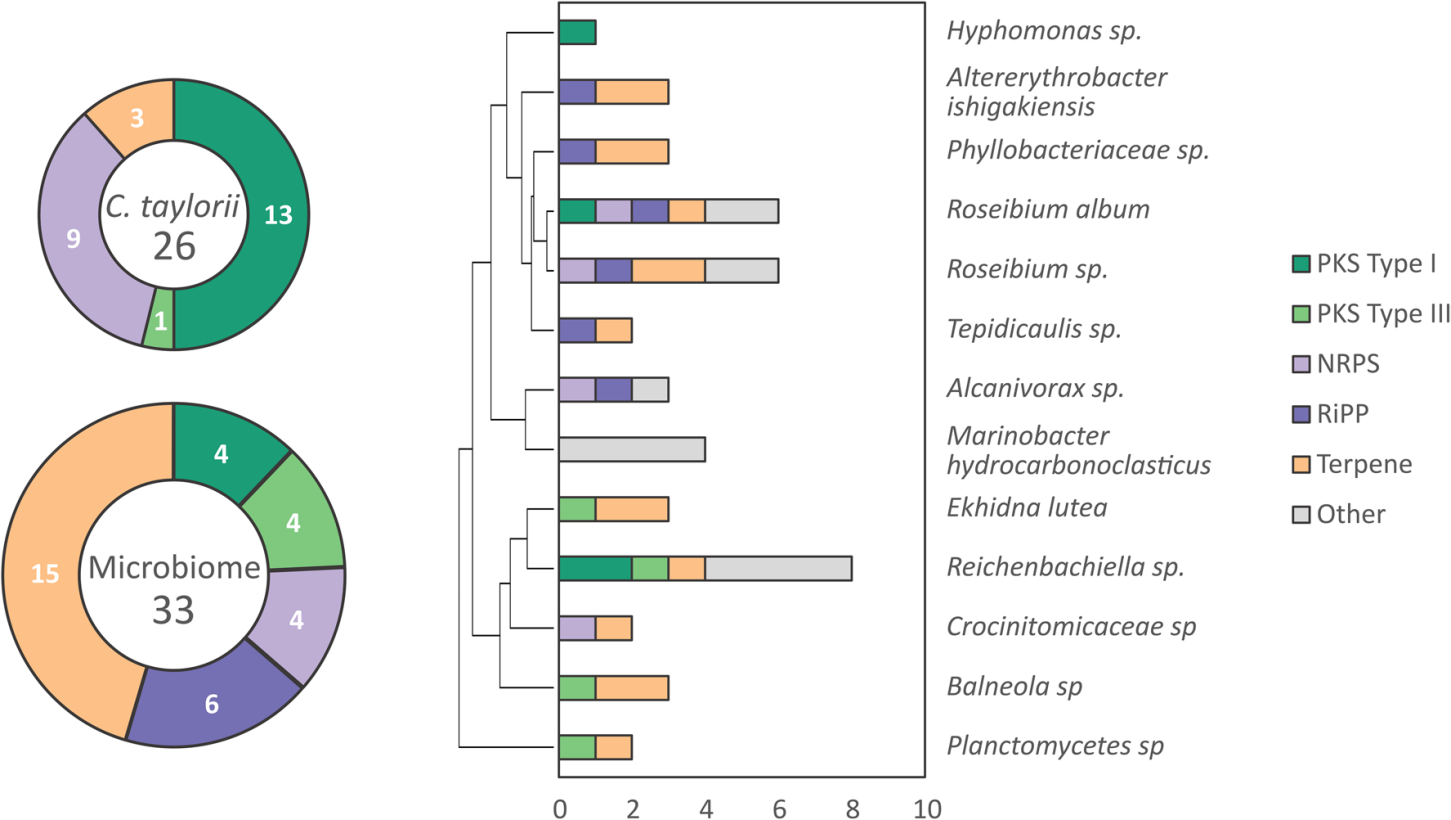
Secondary metabolite gene clusters in *C. taylorii* and its microbiome. Analysis of secondary metabolic gene clusters identified by antiSMASH in *C. taylorii* and its assembled bacterial microbiome (total 26 and 33, respectively, excluding uncategorized clusters). Clusters are divided into the categories type I and III polyketide synthase (PKS), non-ribosomal peptide synthetase (NRPS), ribosomally-encoded post-translationally modified peptide (RiPP), terpene synthase, and other (uncategorized). Bacterial species are organized by hierarchical clustering of 16S sequences.

The microbiome of laboratory-cultured *C. taylorii* was assessed for its secondary metabolic potential (Supplementary Table S2). Each of the assembled bacterial genomes was analyzed by antiSMASH, revealing that most of the bacteria associated with laboratory-cultured *C. taylorii* had a very limited secondary metabolome (Fig. 3). Compared to *C. taylorii*, the microbiome was rich in terpene gene clusters, but significantly limited in polyketide diversity, with only four type I and four type III PKS clusters identified among 13 assemblies. From this analysis, the microbiome of *C. taylorii* was not expected to be the major source of polyketide diversity in *C. taylorii* samples.

**Figure 4:**
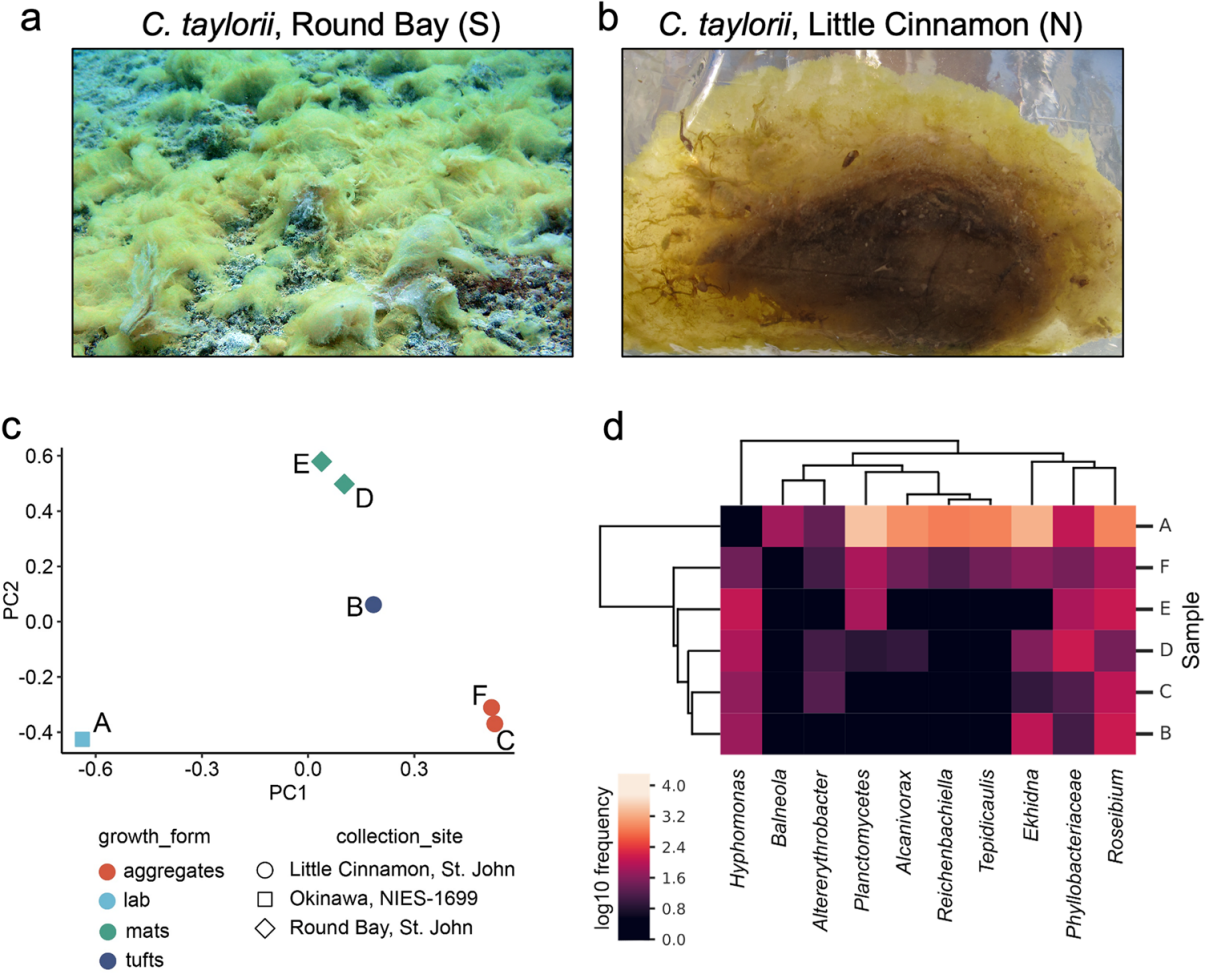
Microbiome of *C. taylorii*. Images of *C. taylorii* colonies with different morphologies collected from (**a**) Round Bay, and (**b**) Little Cinnamon, US Virgin Islands. Colonies consist of yellow *C. taylorii* cells embedded within secreted mucilage. (**c**) Principal component analysis of 16S amplicons from *C. taylorii* samples. (**d**) Heatmap showing the abundance of 16S OTUs assigned to genera common to chrysophaentin-containing samples of (A) laboratory cultivated or (B-F) wild-collected samples of *C. taylorii*. Samples and genera are sorted by hierarchical clustering of the abundance matrix.

The bacterial microbiome of laboratory-cultured *C. taylorii* was compared against five chrysophaentin-producing samples of *C. taylorii* collected from the US Virgin Islands at different sites between 2009 and 2017 (Supplementary Figure S4). The microbiomes of both laboratory-cultured and field collected specimens are composed of three dominant phyla: Bacteroidetes, Proteobacteria, and Planctomycetes. Alpha diversity was lowest in the laboratory-cultured sample. One of the specimens, collected on the southeastern side of St. John at a depth of 15–30′ had the most diverse microbiome with members of the phyla Verrumicrobiota and Firmicutes represented only in this specimen. Samples collected from the south side of the island had a filamentous growth morphology forming mats that spread across many square meters of sand and coral rubble substrate (Fig. 4a). In contrast samples collected on the north side of the island were only observed in high water flow locations and were mostly observed forming mucilaginous aggregates^7^ rather than mats (Fig. 4b) while one sample was tuft-forming^32^. Principal component analysis of the microbiome profiles clearly revealed clustering by growth morphology and sample location suggesting the *C. taylorii* microbiome is shaped by its growth form and environment (Fig. 4c).

The laboratory sample, as expected, was dominated by the bacterial species identified during genome assembly. The same species were also identified in wild collections, but only two taxa, *Labrenzia spp.* and *Phyllobacteriaceae spp.*, were found at significant levels in all samples analyzed (Fig. 4d). Given that chrysophaentins were detected in both laboratory and all wild strains, these taxa are candidates for a possible bacterial origin of the compounds. However, BGC analysis of the genomic assemblies of *Labrenzia spp.* and *Phyllobacteriaceae spp.* revealed that they are particularly poor in PKS gene cluster diversity, with only a single type I PKS observed between the 3 species, and no type III PKS. These data further support an algal origin for the chrysophaentins observed in *C. taylorii* samples.

## Discussion

**Figure 5:**
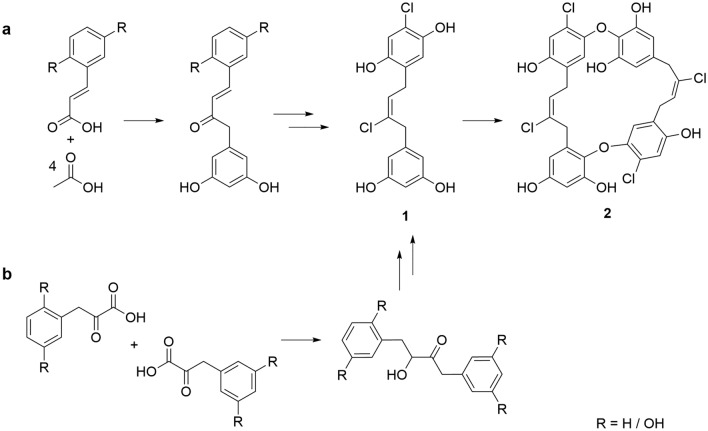
*Biosynthetic proposal for* chrysophaentin A. Two hypothetical biosynthetic routes to chrysophaentin A **2**, via hemichrysophaentin A **1**, utilizing either (**a**) a type III polyketide synthase pathway or (**b**) an acyloin synthase pathway.

The biosynthesis of bisbibenzyl compounds in liverworts is proposed to be controlled by a type III polyketide synthase system that produces a bibenzyl moiety from a cinnamic acid precursor, derived from the shikimate pathway^33^. The bibenzyl intermediate is oxidatively cyclized by a cytochrome P450 to form the bisbibenzyl scaffold^34^. A similar pathway could be envisaged in the production of the chrysophaentins, where a cinnamic acid derivative is extended four times by a type III PKS and tailored to produce hemichrysophaentin **1**, a known metabolite of *C. taylorii* (Fig. 5)^4^. The polyketide cyclization pattern that would produce **1** is typical of the stilbene synthase (STS) clade of type III PKS^35^. Though the chain length differs from wild-type STS enzymes characterized in plants^36^, protein engineering of *Vitis vinifera* STS has been demonstrated to produce homologated bibenzyl products with a core structure analogous to **1**^37^. Radical phenolic coupling, catalyzed by a cytochrome P450 for example^38^, would yield the macrocyclic chrysophaentin metabolites observed in extracts of *C. taylorii*, such as chrysophaentin A **2**. An alternative pathway that could produce the butyl bibenzyl linker of **1** is comparable to the biosynthesis of acyloin metabolites such as the cyanobacterial natural product scytonemin^39^. Condensation of two alpha-ketoacid derivatives of phenylalanine via a TPP-mediated reaction would give the correct scaffold but would require additional tailoring steps to arrive at the putative intermediate hemichrysophaentin A **1** (Fig. 5b).

Type III PKS genes are distributed amongst the genomes of related Stramenopiles algae such as *A. anophagefferens* and *E. siliculosus*. In *E. siliculosus*, there are three PKS genes of this clade. One isoform of the type III PKS in *E. siliculosus* was characterized by heterologous expression, and found to control phlorotannin biosynthesis, a primary metabolite found in brown algal cell walls derived from condensation of four acetate units^40^. The *C. taylorii* genome was found to contain two genes with conserved domains similar to the type III PKS family. One of these is a homolog of the *E. siliculosus* phloroglucinol synthase (61.4% amino acid identity), and fits into the bacterial clade of type III PKS that acts on simple, non-aromatic precursors^36^, and therefore is unlikely to encode the chemistry proposed in Fig. 5 (Supplementary Figure S5). A second *C. taylorii* gene is most similar to the plant fatty acid elongase family (36% amino acid identity, *A. thaliana* KCS-5), rather than typical type III PKS, and would also be expected to act on aliphatic precursors (Supplementary Figure S5)^41^. Based on this analysis, no genes with significant similarity to a stilbene synthase clade gene with an aromatic substrate specificity consistent with the scheme shown in Fig. 5a could be identified in *C. taylorii*. Analysis of the assembled bacterial metagenome of *C. taylorii* identified four species each having a single type III PKS (Fig. 3); however these genes encoded typical bacterial-clade synthases and as such were expected to utilize non-aromatic starter units (Supplementary Figure S5).

Acyloin secondary metabolites of the type detailed in Fig. 5b are biosynthesized by enzymes of the thiamine pyrophosphate (TPP)-dependent acetohydroxyacid synthase (AHAS) superfamily. The *C. taylorii* chromosomal assembly contains two genes with a conserved TPP-binding domain, one of which is a homologue of menD and might participate in the biosynthesis of menaquinone-like molecule^42^. A second enzyme is more similar to benzoyl formate decarboxylase (BFD) that can form benzoin and could conceivably participate in chrysophaentin biosynthesis as described in Fig. 5b (Supplementary Figure S6). Of the two acyloin synthase genes, the BFD-like gene is expressed more strongly than the menD-like gene, though both genes have putative orthologs within other Pelagophyte algae, so are likely to participate in metabolism common within this clade (Fig. 2b).

While the complement of type III PKS genes in the *C. taylorii* genome is modest, an expanded set of type I PKS genes was identified. A feature of the majority of PKS genes in *C. taylorii* is the presence of an N-terminal adenylation domain capable of loading advanced substrate precursors. Such N-terminal domains have been observed in other algal PKS genes, but to date none have been functionally characterized^43^. This domain could allow the synthase to utilize advanced precursors such as cinnamate, mimicking the reactivity of a plant type III PKS and potentially facilitating the pathway detailed in Fig. 5a. One candidate in particular contains four extension modules without reductive domains and may be capable of this reaction (Fig. 2a).

## Conclusions

A bloom-forming, and chrysophaentin-producing sample of *C. taylorii* was genomically characterized using short and long read technologies, revealing a core algal contig set comprising 71 Mbp and 10,810 high confidence predicted genes, alongside a small microbiome comprising at least 13 distinct bacterial species. Principal component clustering of the microbiomes associated with geographically and morphologically distinct samples collected around St. John, USVI, indicate these factors shape the *C. taylorii* microbiome. Interestingly, the morphologies are consistent with those observed in low flow vs high flow waters and described by Caronni et al.^7^. *C. taylorii* contained an expanded complement of secondary metabolic genes compared to its closest relative *A. anophagefferens*, reflecting its greater chemical diversity of natural product biosynthesis, including the chrysophaentins. However, no type III PKS with homology to the aryl-extending plant-type chalcone-synthase clade was identified in the genome, which we had hypothesized to be necessary for chrysophaentin biosynthesis. Instead, a highly diverse and non-canonical complement of PKS genes was identified in the chrysophaentin producer, many of which contained an N-terminal adenylation domain that could be responsible for loading advanced starter units such as the cinnamic acid derivative expected to be involved in chrysophaentin biosynthesis (Fig. 5a). While polyketide biosynthesis in stramenopiles algae is still poorly characterized, unusual PKS genes have been noted in the genomes of *Emiliana huxleyi* and *A. anophagefferens*, as well as in symbiotic dinoflagellates^44–46^. Further characterization of this intriguing branch of polyketide biosynthesis will require the development of more advanced molecular genetic tools in the stramenopiles algae, and awaits more thorough genomic exploration of this sparsely sampled clade of microbial organisms. Nevertheless, the sequencing and analyses described here substantially expand the genomic landscape for the Pelagophyte algae, an important group owing to their sheer numerical abundance and the ecological effects members of this group exert on many marine environments.

## Methods

### Sampling and sequencing

Field-collected samples of *Chrysophaeum taylorii* were made in St. John, U.S. Virgin Islands at the sites listed in Supplementary Figure S4. During collection, *C. taylorii* samples were gently removed from substrate, and extraneous debris was gently removed while under water. Samples were collected in plastic bags with sea water present, and voucher specimens were immediately frozen or placed in RNAlater or 70% EtOH and stored at 4 °C. *Chrysophaeum taylorii* NIES-1699 was obtained from the microbial culture collection at the Japanese National Institute for Environmental Studies (NIES). The strain was originally collected from Iriomote Island, Okinawa, Japan. Cultures were grown in MNK enriched seawater medium at 25 °C with shaking at 90 rpm, illuminated on a 12 h/12 h light dark cycle for up to 8 weeks before harvest; media was replaced with fresh stock every 2 weeks. Colonial cell mass was harvested with a sterile hook. Associated bacterial populations were controlled before sample extraction by exposure to an antibiotic cycle of kanamycin (200 ug/mL, 2 days), followed by penicillin (100 U/mL)/streptomycin (100 ug/mL) (2 days), then erythromycin (20 ug/mL, 2 days).

Illumina DNA sequencing libraries were prepared by extraction of nucleic acids from *C. taylorii* colonies via a protocol developed for mucilaginous samples of *Ectocarpus siliculosus*^47^. Two DNA libraries were prepared for Illumina 150 bp paired end sequencing with insert sizes of average length 400 and 850 bp by shearing gDNA samples on a Covaris S220 focused ultrasonicator, followed by library preparation with the NEBNext Ultra II DNA kit (NEB). The two libraries were combined in an equimolar ratio and sequenced on two lanes of a HiSeq 2500 (Illumina). RNA samples were extracted with the Rneasy mini kit (Qiagen), and libraries were prepared for Illumina 50 bp single end sequencing with an average insert size of 400 bp by selection of mRNA using the NEBNext Poly(A) mRNA Magnetic Isolation Module (NEB) followed by NEBNext Ultra II Directional RNA Library Prep Kit (NEB). RNA libraries were sequenced on a HiSeq 2500 (Illumina).

Genomic DNA was prepared for long read sequencing by extraction in Carlson CTAB buffer at 65 °C^48^, followed by anion-exchange purification of isopropyl alcohol precipitated nucleic acids on a Genomic-Tip 20 column (Qiagen). Sequencing libraries were prepared with the SQK-LSK108 ligation kit (Oxford Nanopore) and run on three FLO-MIN106 MK1 R9 flowcells. Additionally, one library was prepared by whole genome amplification of gDNA using the REPLI-g midi kit (Qiagen) followed by SQK-LSK108 ligation kit, to reduce sample contamination before sequencing on one FLO-MIN106 MK1 R9 flowcell. Genomic DNA was also used to prepare one library with the SMRTbell kit for SEQUEL sequencing (Pacific Biosciences) on 8 cells.

## Supplementary Information

Supplementary Information.

## Data Availability

All sequencing data sets generated and analyzed in this study have been deposited under NCBI Project accession number PRJNA892039. The assembled genome sequence is available via NCBI GenBank (accession: JAQMWT000000000). 16S microbiome data can be found under the sequence read archive accession numbers SRR22085502-SRR22085507. Illumina WGS data can be retrieved from SRR22085760 and RNA-sequencing data from SRR22085759. PacBio WGS data with SRR22085545.
