# Supplementary material for: The genome of antibiotic-producing colonies of the Pelagophyte alga Chrysophaeum taylorii reveals a diverse and non-canonical capacity for secondary metabolism
Source: Sci Rep. 2023 Jul 24;13:11944. doi: 10.1038/s41598-023-38042-8 (PMC10366177; doi:10.1038/s41598-023-38042-8)
Supplement: Supplementary file 1 — Supplementary Information. [file 41598_2023_38042_MOESM1_ESM.docx]

Supplementary material

**Supplementary Methods**

[Supplementary Methods 2](#_Toc129704588)

[Genome assembly and annotation 2](#_Toc129704589)

[Phylogenetic analysis 3](#_Toc129704590)

[Microbiome analysis 3](#_Toc129704591)

[Supplementary Tables 4](#_Toc129704592)

[Supplementary Figures 10](#_Toc129704593)

**Supplementary Tables**

[Supplementary Table S 1: Summary statistics of raw sequencing data 5](#_Toc129704594)

[Supplementary Table S 2: Taxonomic assignment of bacterial metagenome-assembled genomes 6](#_Toc129704595)

[Supplementary Table S 3: Assembly refinement statistics. 7](#_Toc129704596)

[Supplementary Table S 4: Gene kinds in BGC - C. taylorii vs microbiome. 8](#_Toc129704597)

[Supplementary Table S 5: Number of halogenases in C. taylorii genome. 9](#_Toc129704598)

**Supplementary Figures**

[Supplementary Figure S 1: Comparison of genome sequence annotation and repeat content between *C. taylorii* and related algae. 11](#_Toc129704599)

[Supplementary Figure S 2: Organization of biosynthetic core and tailoring genes in antiSMASH predicted BGCs in *C. taylorii.* 12](#_Toc129704600)

[Supplementary Figure S 3: Number of genes in BGCs in Cyanobacteria. 13](#_Toc129704601)

[Supplementary Figure S 4: 16S microbiome analysis of laboratory and wild *C. taylorii* strains. 14](#_Toc129704602)

[Supplementary Figure S 5: Phylogenetic placement of *C. taylorii* type-III polyketide synthase. 15](#_Toc129704603)

[Supplementary Figure S 6: Phylogenetic placement of *C. taylorii* acyloin synthase-like proteins together with other previously known thiamine pyrophosphate (TPP) binding proteins. 16](#_Toc129704604)

# Supplementary Methods

## ****Genome assembly and annotation****

Raw illumina sequencing reads were processed by adapter trimming and contaminant filtering (BBduk: BBtools suite version 38.57) followed by merging overlapping pairs (BBmerge: BBtools suite version 38.57). Reads were assembled using metaSPAdes (SPAdes version 3.13.0), with merged reads submitted as a single-ended library, and the remaining reads submitted as pairs. Eukaryotic contigs were separated from bacterial using Eukrep (version 0.6.6). Distinct genomes were deconvoluted from the assembly using Metabat (version 2.12.1) as well as by manual inspection of the assembly graph using Bandage (version 0.8.1). Assembly quality was assessed using BUSCO (version 3.0.2) in comparison to the Eukaryota_ODB8 dataset. Reads were aligned against assemblies using BBmap (BBtools suite version 38.57) for short reads or minimap2 (version 2.11) for long reads. RNAseq data was assembled de novo using Trinity (version 2.6.5), and aligned to genomic assemblies using TopHat (version 2.1.1). PacBio reads were assembled using Falcon and polished using Arrow from the PacBio SMRT Tools suite (version 6.0.0), while Nanopore reads were basecalled using Guppy (version 2.3.5) assembled using Canu (version 1.8) and polished using Medaka (version 6.0.1) and Pilon (version 1.23).

The final *C. taylorii* genome assembly was prepared by initial binning of long reads by alignment to eukaryotic chromosomal contigs from metaSPAdes Illumina read assembly (after removal of contigs assigned as bacteria, mitochondria, or chloroplast). Binned eukaryotic reads were assembled using Canu, polished using Medaka, and subjected to a 6-step assembly refinement pipeline. 1) removal of mitochondrial or chloroplast contigs by manual inspection of high coverage contigs using BLAST, 2) filtering by coverage > 10 and contig length > 15 kb (2x median read length), 3) removal of heterozygous or redundant repeat contigs using the purge_haplotigs software package (version 1.0.4), 4) assembly polishing using Pilon and Racon with Illumina reads, 5) filtering of contigs with abnormal coverage of Illumina DNA- and RNA-seq reads, 6) removal of remaining bacterial contigs by manual inspection in comparison to sequence databases. Table S5 details assembly statistics for each refinement step; % RNA-seq reads aligned and BUSCO analysis were used to assess the maintenance of coding sequence as redundant and repetitive contigs were filtered from the assembly.

**Gene annotations for bacterial assemblies were made using Prokka (version 1.13), and for chloroplast and mitochondrion using GeSeq (version 2.03). Annotations for the *C. taylorii* genome were made using the Braker2 and Maker2 pipelines. Braker2 was used to train initial gene predictions for Augustus (version 3.2.2 ) using intron models from RNA-seq alignments. The Augustus training model was used for *de novo* prediction in Maker2 alongside protein homology evidence from five related algal NCBI proteomes (*Aureococcus anophagefferens, Nannochloropsis gaditana, Ectocarpus siliculosis, Phaeodactylum tricornatum, Thassalonicum pseudomarin*) in addition to the UniProt database of curated proteins and transcript evidence from *de novo* assembled RNA-seq data. antiSMASH 6.0.0 was run in both fungal and bacterial mode on the final assembled and annotated genome to identify secondary metabolic genes. Both fungal and bacterial software variants gave identical output.**

## ****Phylogenetic analysis****

**Taxonomic assignments of bacterial assemblies were made by extraction of 16S sequences from each genome, followed by BLAST analysis using 16 rRNA sequence as query and NCBI RefSeq curated 16S rRNA records as database. The taxonomy of the most closely related sequence in the database (i.e., highest percent identity and lowest by E-value) was assigned to the assembly.**

**Phylogenetic analysis of *C. taylorii* was performed by concatenation of the 18S rRNA sequence with the mitochondrial genes psaA, psaB, psbA, psbC, and rbcL and alignment against the orthologous sequences from a set of pelagophyte algae. The trimmed alignment was used to build a maximum likelihood phylogenetic tree using RAxML (8.2.11) with the GTR gamma model, with 1000 rapid bootstrap replicates. Additionally, a Bayesian phylogenetic tree was constructed by MrBayes (3.2.6) using the GTR gamma model, a chain length of 1000000 with burn-in length 100000. The topologies of the trees derived from both methods were equivalent to each other and similar to previously published phylogenies. Bootstrap support values and posterior probability values were calculated to provide support to the inferred clades.**

## ****Microbiome analysis****

***C. taylorii* microbiome analysis was performed on DNA extracts sampled from laboratory cultures that underwent genome sequencing, as well as on samples that had been collected from locations surrounding St. John, US Virgin Islands in 2009 and 2017 and preserved in RNAlater at 4 °C. The 16S V3-V4 sequence was amplified using universal primer sequences TCGTCGGCAGCGTCAGATGTGTATAAGAGACAGCCTACGGGNGGCWGCAG and GTCTCGTGGGCTCGGAGATGTGTATAAGAGACAGGACTACHVGGGTATCTAATCC with Illumina adapters included. Sequencing libraries were prepared and sequenced by MiSeq 250 bp PE reads. Read pairs were merged using** BBduk (BBtools suite version 38.57) and analyzed by Qiime2 (2019.1.0) using the Deblur plugin for denoising, and taxonomic analysis using the Greengenes database.

# Supplementary Tables

Supplementary Table S 1: Summary statistics of raw sequencing data

| Technnology | layout | Platform | paired  end | additional_info | n_reads | n_bases | readlenN50 | readlenAvg | readlenMax |
| --- | --- | --- | --- | --- | --- | --- | --- | --- | --- |
| Illumina | WGS | HiSeq | Y | lane1-400bp-insert | 61360531 | 18530880362 | 151 | 151 | 151 |
| Illumina | WGS | HiSeq | Y | lane2-400bp-insert | 63144338 | 19069590076 | 151 | 151 | 151 |
| Illumina | WGS | HiSeq | Y | lane1-850bp-insert | 54002815 | 16308850130 | 151 | 151 | 151 |
| Illumina | WGS | HiSeq | Y | lane2-850bp-insert | 55290794 | 16697819788 | 151 | 151 | 151 |
| Illumina | RNA-seq | HiSeq | N | Qia-250-insert | 30206598 | 1540536498 | 51 | 51 | 51 |
| Illumina | RNA-seq | HiSeq | N | Qia-800-insert | 28298900 | 1443243900 | 51 | 51 | 51 |
| Illumina | RNA-seq | HiSeq | N | Greco-250-insert | 31710043 | 1617212193 | 51 | 51 | 51 |
| Illumina | RNA-seq | HiSeq | N | Greco-800-insert | 30260991 | 1543310541 | 51 | 51 | 51 |
| PacBio | WGS | Sequel | N | SmrtCell_1 | 593681 | 4679010716 | 9426 | 7882 | 82915 |
| PacBio | WGS | Sequel | N | SmrtCell_2 | 780803 | 6233175493 | 9380 | 7984 | 74785 |
| PacBio | WGS | Sequel | N | SmrtCell_3 | 867750 | 6594943910 | 9295 | 7601 | 91626 |
| PacBio | WGS | Sequel | N | SmrtCell_4 | 1572600 | 13921119077 | 10009 | 8853 | 177450 |
| PacBio | WGS | Sequel | N | SmrtCell_5 | 1526168 | 12556213942 | 9815 | 8228 | 118479 |
| PacBio | WGS | Sequel | N | SmrtCell_6 | 590356 | 4524043212 | 9268 | 7664 | 76332 |
| PacBio | WGS | Sequel | N | SmrtCell_7 | 992328 | 7769498561 | 9409 | 7830 | 79634 |
| PacBio | WGS | Sequel | N | SmrtCell_8 | 682499 | 5181416151 | 9368 | 7592 | 77671 |
| Nanopore | WGS | MinION | N | Combined Nanopore | 8052400 | 39349899722 | 6265 | 4887 | 123028 |
| Illumina | 16S amplicon | MiSeq | Y | sampleA | 1294802 | 649990604 | 251 | 251 | 251 |
| Illumina | 16S amplicon | MiSeq | Y | sampleB | 1343664 | 674519328 | 251 | 251 | 251 |
| Illumina | 16S amplicon | MiSeq | Y | sampleC | 1132528 | 568529056 | 251 | 251 | 251 |
| Illumina | 16S amplicon | MiSeq | Y | sampleD | 822024 | 412656048 | 251 | 251 | 251 |
| Illumina | 16S amplicon | MiSeq | Y | sampleE | 1724910 | 865904820 | 251 | 251 | 251 |
| Illumina | 16S amplicon | MiSeq | Y | sampleF | 1168185 | 586428870 | 251 | 251 | 251 |

Supplementary Table S 2: Taxonomic assignment of bacterial metagenome-assembled genomes

| Taxonomy | pident | closest_ncbi_gi | phylum | class | order | family | genus | species |
| --- | --- | --- | --- | --- | --- | --- | --- | --- |
| Marinobacter nauticus | 99.935 | 444439468 | Proteobacteria | Gammaproteobacteria | Pseudomonadales | Marinobacteraceae | Marinobacter | *Marinobacter nauticus* |
| Ekhidna lutea | 99.829 | 636559004 | Bacteroidetes | Cytophagia | Cytophagales | Reichenbachiellaceae | Ekhidna | *Ekhidna lutea* |
| Alcanivorax jadensis | 98.999 | 219878132 | Proteobacteria | Gammaproteobacteria | Oceanospirillales | Alcanivoracaceae | Alcanivorax | *Alcanivorax jadensis* |
| Hyphomonas beringensis | 98.706 | 1212229199 | Proteobacteria | Alphaproteobacteria | Hyphomonadales | Hyphomonadaceae | Hyphomonas | *Hyphomonas beringensis* |
| Roseibium album | 98.607 | 343201652 | Proteobacteria | Alphaproteobacteria | Hyphomicrobiales | Stappiaceae | Roseibium | *Roseibium album* |
| Altererythrobacter ishigakiensis | 98.371 | 631251524 | Proteobacteria | Alphaproteobacteria | Sphingomonadales | Erythrobacteraceae | Altererythrobacter | *Altererythrobacter ishigakiensis* |
| Roseibium sp. | 97.729 | 343201652 | Proteobacteria | Alphaproteobacteria | Hyphomicrobiales | Stappiaceae | Roseibium | *Roseibium album* |
| Reichenbachiella sp. | 96.675 | 645320177 | Bacteroidetes | Cytophagia | Cytophagales | Reichenbachiellaceae | Reichenbachiella | *Reichenbachiella faecimaris* |
| Tepidicaulis sp. | 96.033 | 1013172398 | Proteobacteria | Alphaproteobacteria | Hyphomicrobiales | Parvibaculaceae | Tepidicaulis | *Tepidicaulis marinus* |
| Balneola sp. | 95.36 | 343202612 | Balneolaeota | Balneolia | Balneolales | Balneolaceae | Balneola | *Balneola vulgaris* |
| Phyllobacteriaceae sp. | 94.622 | 959494960 | Proteobacteria | Alphaproteobacteria | Hyphomicrobiales | Phyllobacteriaceae | Phyllobacterium | *Phyllobacterium loti* |
| Crocinitomicaceae sp. | 92.875 | 631251782 | Bacteroidetes | Flavobacteriia | Flavobacteriales | Crocinitomicaceae | Salinirepens | *Salinirepens amamiensis* |
| Planctomycetes sp. | 83.569 | 1270533064 | Planctomycetes | Phycisphaerae | Phycisphaerales | Phycisphaeraceae | Phycisphaera | *Phycisphaera mikurensis* |

Supplementary Table S 3: Assembly refinement statistics.

| Refinement Step | Assembly Size / Mbp | Number of contigs | N50 / kbp | % RNA-seq alignment | BUSCOs / % |
| --- | --- | --- | --- | --- | --- |
| 0 | 132.5 | 4008 | 56.7 | 88.7 | 70.3 |
| 1 | 131.1 | 3959 | 57.4 | 88.4 | 70.3 |
| 2 | 98.7 | 1539 | 97.0 | 87.3 | 70.0 |
| 3 | 76.9 | 888 | 135.7 | 85.8 | 70.0 |
| 4 | 73.7 | 765 | 146.2 | 87.2 | 81.8 |
| 5 | 71.2 | 705 | 157.4 | 86.8 | 81.2 |
| 6 | 71.0 | 702 | 157.4 |  |  |

Supplementary Table S 4: Gene kinds in BGC - C. taylorii vs microbiome.

|  | C. taylorii | | | microbiome | | |
| --- | --- | --- | --- | --- | --- | --- |
| gene_kind | avg_per_region | min_per_region | max_per_region | avg_per_region | min_per_region | max_per_region |
| Core | 1 | 1 | 1 | 1.47 | 1 | 5 |
| Other | 4.12 | 0 | 12 | 15 | 2 | 36 |
| Regulatory | 0.0769 | 0 | 1 | 1.16 | 0 | 6 |
| Tailoring | 1.19 | 0 | 3 | 4.24 | 0 | 14 |
| Transport | 0.154 | 0 | 1 | 0.959 | 0 | 7 |

Supplementary Table S 5: Number of halogenases in *C. taylorii* genome.

| domain_id | domain_short | domain_full | gene_count |
| --- | --- | --- | --- |
| PF04820 | Trp_halogenase | Tryptophan halogenase | 0 |
| PIRSF011396 | Trp_halogenase | Tryptophan halogenase | 0 |
| IPR006905 | Flavin_halogenase | Flavin-dependent halogenase | 0 |
| IPR033856 | Trp_halogen | Flavin-dependent tryptophan halogenase | 0 |
| PTHR43747:SF5 | HALOGENASE-RELATED | HALOGENASE-RELATED | 0 |
| PTHR43747:SF4 | A0A143CDU1_9ACTN | A0A143CDU1_9ACTN | 0 |
| PF00561 | Abhydrolase_1 | alpha/beta hydrolase fold | 14 |
| PF17897 | VCPO_N | Vanadium chloroperoxidase N-terminal domain | 0 |
| PF01569 | PAP2 | PAP2 superfamily | 8 |
| PF01328 | Peroxidase_2 | Peroxidase, family 2 | 0 |
| PF05721 | PhyH | Phytanoyl-CoA dioxygenase (PhyH) | 32 |
| PF01887 | SAM_HAT_N | SAM hydroxide adenosyltransferase N-terminal domain | 0 |
| PF20257 | SAM_HAT_C | SAM hydroxide adenosyltransferase C-terminal domain | 0 |

# Supplementary Figures


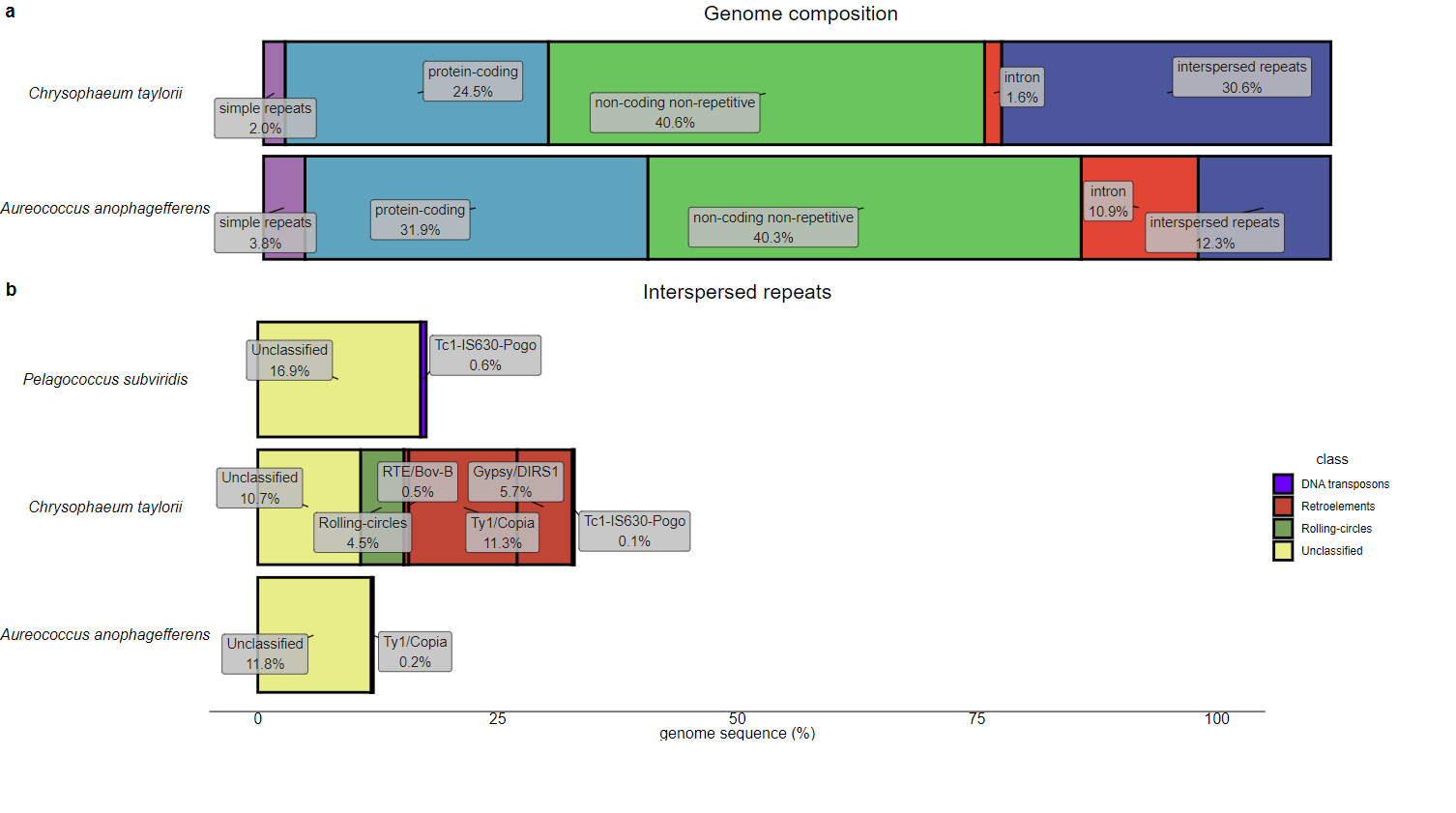


Supplementary Figure S 1: Comparison of genome sequence annotation and repeat content between *C. taylorii* and related algae.

a) Repeat composition of the genome in *C. taylorii* and *A. anophagefferens*. Percentages indicate proportion of specific repeat or coding sequence in total genome sequence. b) Distribution of various interspersed repeat classes in genome of *P. subviridis*, *C. taylorii* and *A. anophagefferens.*


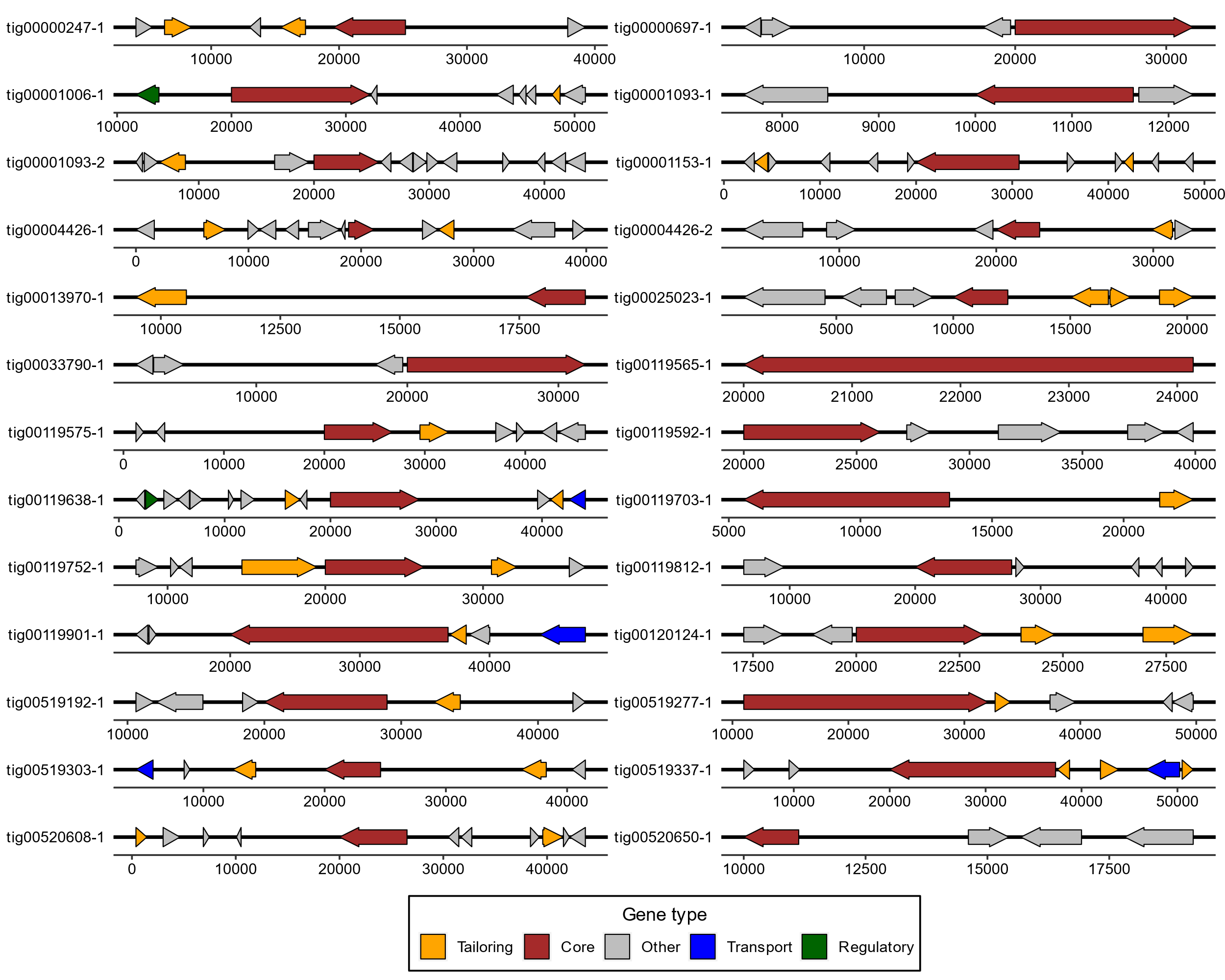


Supplementary Figure S 2: Organization of biosynthetic core and tailoring genes in antiSMASH predicted BGCs in *C. taylorii.*

Gene organization within antiSMASH predicted BGCs is shown. Contig ids are labelled on the y-axis with scale bar indicating position from the start of antiSMASH predicted BGC. Genes are colored according to their biosynthetic function types (core, tailoring, transport or regulatory). ‘Other‘ indicate that the gene had no clearly assigned secondary metabolite related function such as hypothetical proteins.


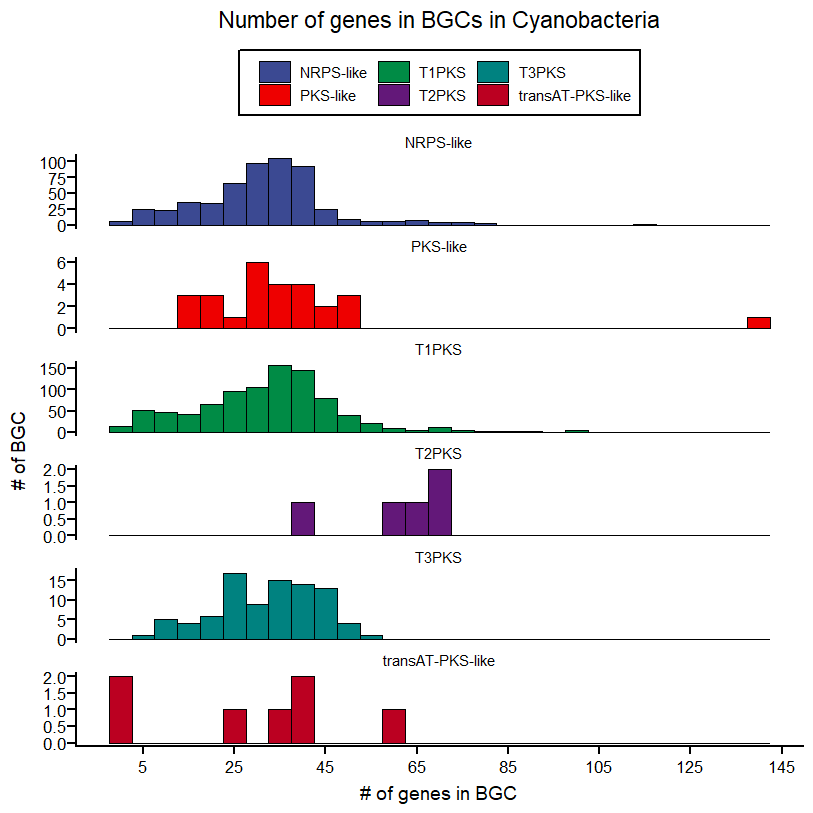


Supplementary Figure S 3: Number of genes in BGCs in Cyanobacteria.

Histograms of number of genes in each BGC type in phylum Cyanobacteria indicating higher number of genes per BGC than observed in *C. taylorii* (this study). The data for the chart was sourced from BiG-FAM database.


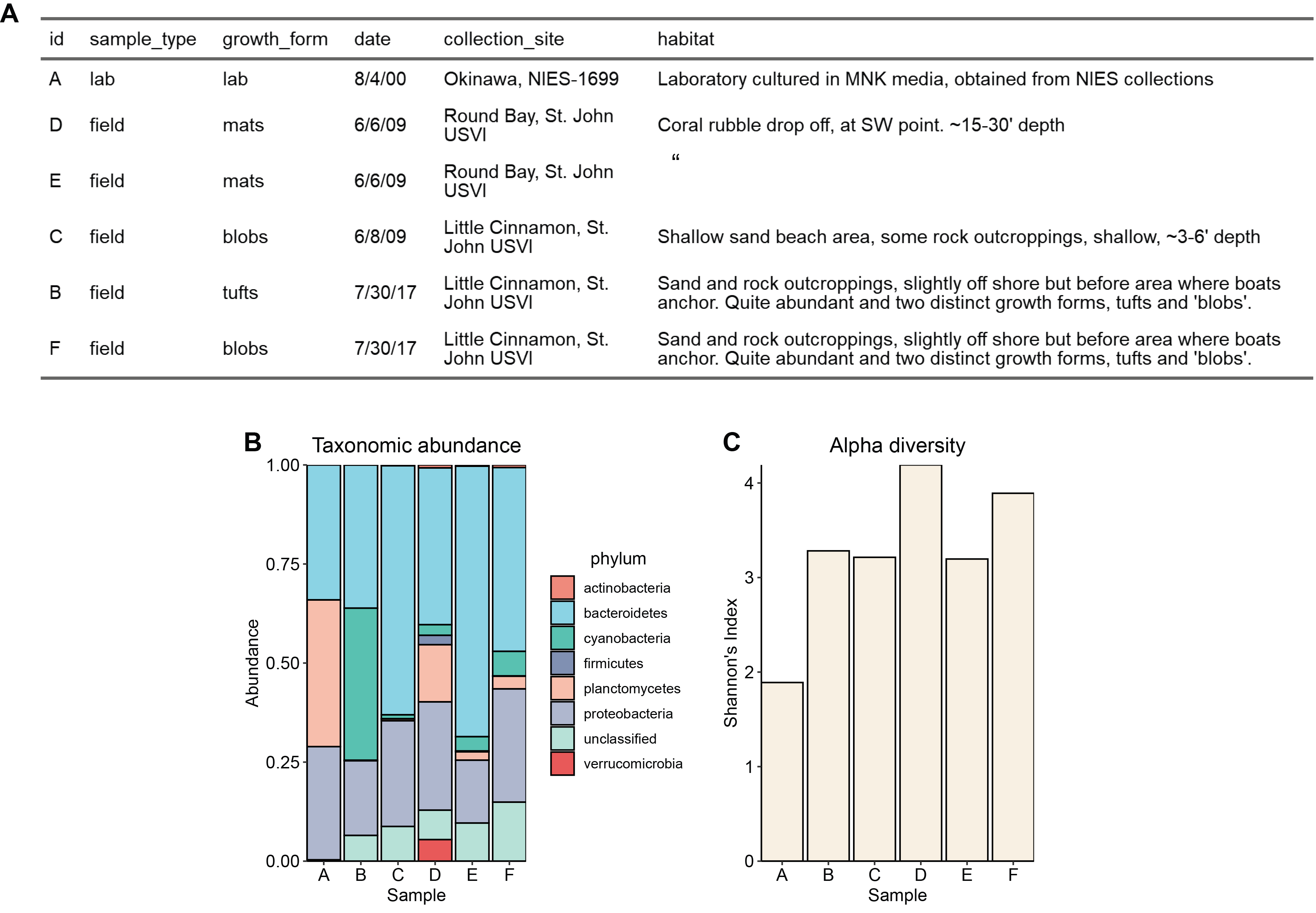


Supplementary Figure S 4: 16S microbiome analysis of laboratory and wild *C. taylorii* strains.

A) Sample IDs, collection sites and growth forms of *C. taylorii* samples used in this study. B) Phylum level composition of associated microbiomes shown by taxonomy bar plot. C) Shannon’s index as a measure of alpha diversity across samples.


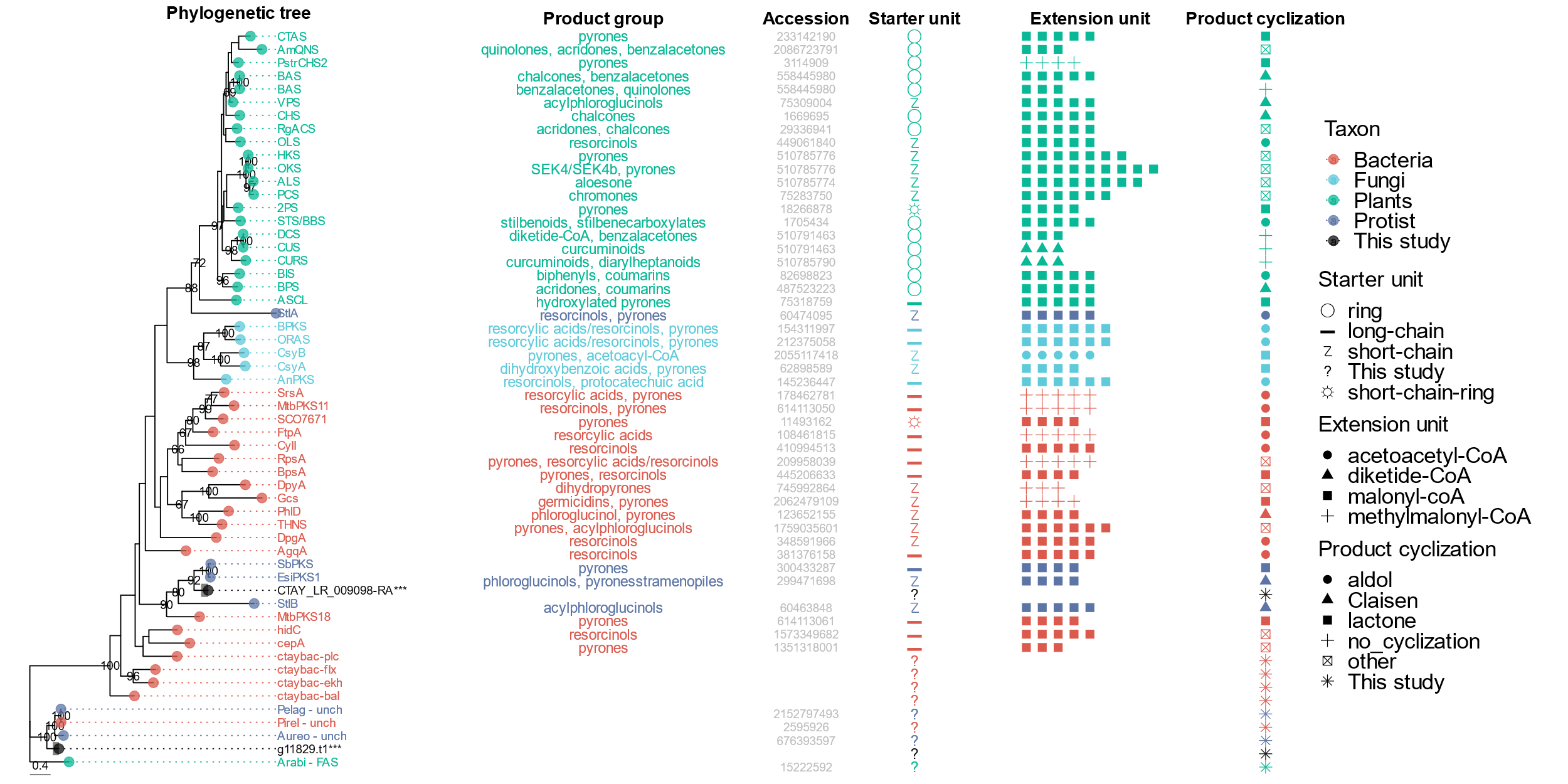


Supplementary Figure S 5: Phylogenetic placement of *C. taylorii* type-III polyketide synthase.

The two polyketide synthases from *C. taylorii* are labelled CTAY_LR_009098-RA and g11829.t1. Bacterial type-iii PKSs from *C. taylorii* microbiome are labelled with ctaybac followed with abbreviated name for organism - plc (Planctomycetaceae), ekh (Ekhidna), flx (Flexibacteriaceae) or bal (Balneola). Most sequences and annotation are derived from PMID: 27862822. A few uncharacterized enzymes (labelled as -unch) with high sequence similarity to g11829.t1 were also included in the analysis. These were from Pelagomonas (Pelag – unch), Aureococcus (Aureo - unch) and Pirellulales (Pirel – unch). The phylogenetic tree is rooted with fatty-acid synthase from Arabidopsis (Arabi – FAS). Node labels indicate clade-support values.


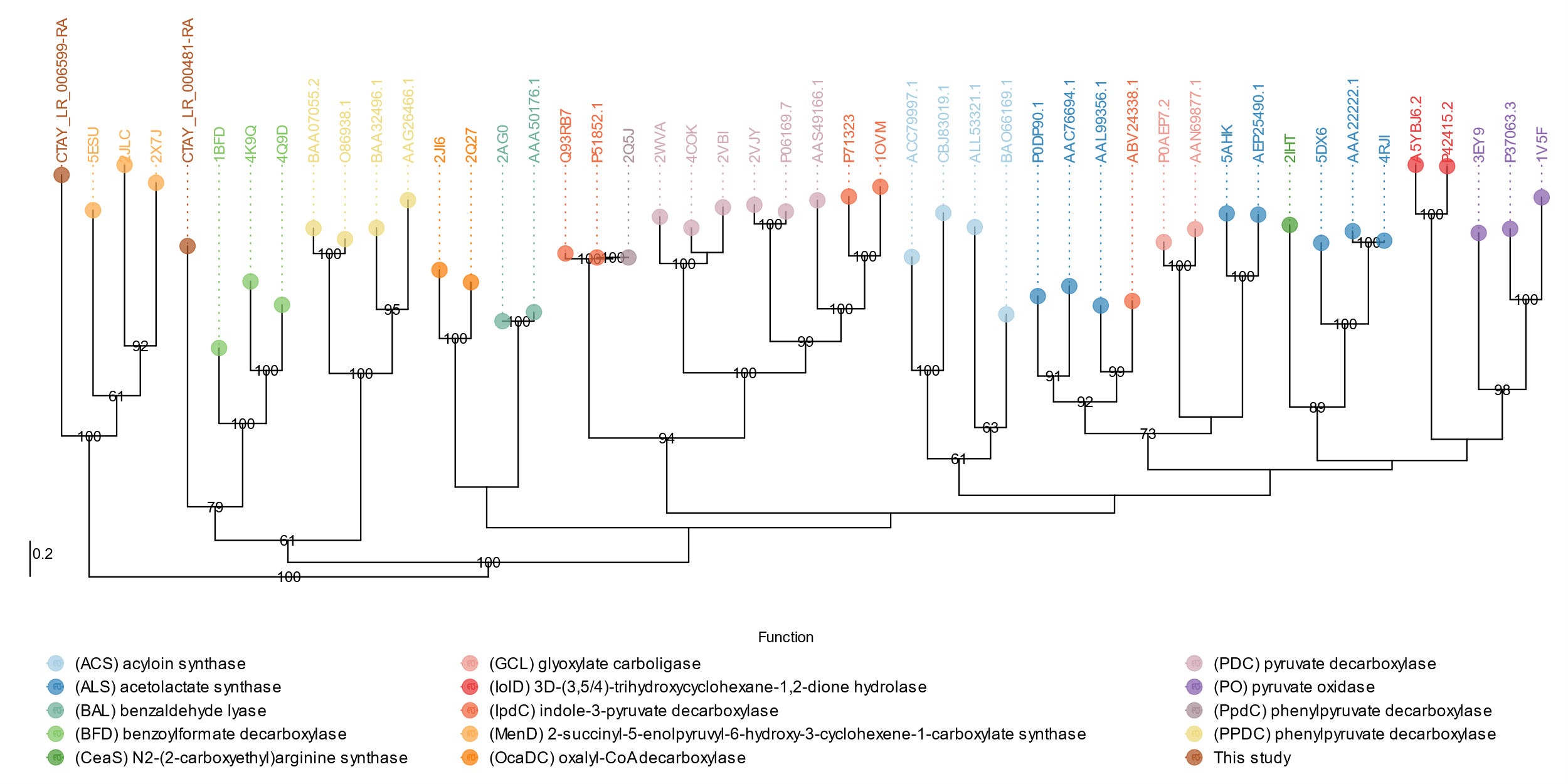


Supplementary Figure S 6: Phylogenetic placement of *C. taylorii* acyloin synthase-like proteins together with other previously known thiamine pyrophosphate (TPP) binding proteins.

*C. taylorii* enzymes are labelled with CTAY_LR* and highlighted in brown color. Node labels indicate clade-support values. Sequences and annotations are derived from PMID: 31243958.
